# Molecular mechanisms governing circulating immune cell heterogeneity across different species revealed by single‐cell sequencing

**DOI:** 10.1002/ctm2.689

**Published:** 2022-01-29

**Authors:** Zhibin Li, Chengcheng Sun, Fei Wang, Xiran Wang, Jiacheng Zhu, Lihua Luo, Xiangning Ding, Yanan Zhang, Peiwen Ding, Haoyu Wang, Mingyi Pu, Yuejiao Li, Shiyou Wang, Qiuyu Qin, Yanan Wei, Jian Sun, Xiangdong Wang, Yonglun Luo, Dongsheng Chen, Wei Qiu

**Affiliations:** ^1^ Department of Neurology The Third Affiliated Hospital of Sun Yat‐Sen University Guangzhou China; ^2^ BGI‐Shenzhen Shenzhen China; ^3^ College of Life Sciences University of Chinese Academy of Sciences Beijing China; ^4^ Department of Biomedicine Aarhus University Aarhus Denmark; ^5^ Lars Bolund Institute of Regenerative Medicine Qingdao‐Europe Advanced Institute for Life Sciences, BGI‐Qingdao, BGI‐Shenzhen Qingdao China; ^6^ National Risk Assessment Laboratory for Antimicrobial Resistance of Animal Original Bacteria South China Agricultural University Guangzhou China; ^7^ Guangdong Laboratory for Lingnan Modern Agriculture Guangzhou China; ^8^ Tsinghua‐Berkeley Shenzhen Institute Tsinghua University Shenzhen China; ^9^ Department of Pulmonary and Critical Care Medicine Zhongshan Hospital Shanghai China; ^10^ Fudan University Shanghai Medical College Shanghai China; ^11^ Steno Diabetes Center Aarhus Aarhus University Hospital Aarhus Denmark

**Keywords:** cross‐species, peripheral blood mononuclear cells, single‐cell RNA sequencing

## Abstract

**Background:**

Immune cells play important roles in mediating immune response and host defense against invading pathogens. However, insights into the molecular mechanisms governing circulating immune cell diversity among multiple species are limited.

**Methods:**

In this study, we compared the single‐cell transcriptomes of immune cells from 12 species. Distinct molecular profiles were characterized for different immune cell types, including T cells, B cells, natural killer cells, monocytes, and dendritic cells.

**Results:**

Our data revealed the heterogeneity and compositions of circulating immune cells among 12 different species. Additionally, we explored the conserved and divergent cellular crosstalks and genetic regulatory networks among vertebrate immune cells. Notably, the ligand and receptor pair VIM‐CD44 was highly conserved among the immune cells.

**Conclusions:**

This study is the first to provide a comprehensive analysis of the cross‐species single‐cell transcriptome atlas for peripheral blood mononuclear cells (PBMCs). This research should advance our understanding of the cellular taxonomy and fundamental functions of PBMCs, with important implications in evolutionary biology, developmental biology, and immune system disorders.

## Graphical abstract

Our study compared the single‐cell transcriptomes of immune cells from 12 species. The results revealed the heterogeneity and compositions of circulating immune cells. We explored the conserved and divergent cellular crosstalks and genetic regulatory networks among vertebrate immune cells.

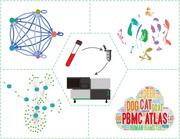

## 1 INTRODUCTION

Peripheral blood mononuclear cells (PBMCs) are derived from myeloid and lymphoid hematopoietic systems and are mainly comprised of circulating multi‐functional immune cell types, such as lymphocytes, monocytes, and dendritic cells (DCs). As the supervisor and executor of body defense, PBMCs play important roles in mediating innate and adaptive immune responses, maintaining immune homeostasis, and reflecting the real‐time cellular and humoral immune state of the whole body. As such, PBMCs are widely used in the fields of immunology,^1^, ^2^ infectious diseases,^3^, ^4^ cancer,^5^ vaccine development,^6^ transplantation^7^ and high‐throughput screening for therapeutic antibodies.^8^ As a commonly used *ex vivo* cellular model in immunological function studies, PBMCs also play a vital role in immunological research and immunotherapy, and have been used to predict diagnostic biomarkers and discover potential immunotherapy targets.^9^, ^10^

Traditional RNA sequencing (RNA‐seq) provides the ability to measure average gene expression of the entire transcriptome from bulk cells, which hides the potential cellular heterogeneity.^11^ Single‐cell RNA‐sequencing (scRNA‐seq) offers an unbiased approach to deconvolve the heterogeneity of immune cells and profile cell breadth (cell number) and depth (gene number per cell).^12^

Due to the complexity of PBMCs, it is difficult to study the function of individual immune cells. However, advances in scRNA‐seq allow comprehensive analysis of the immune system at the single‐cell resolution. scRNA‐seq can capture gene expression of individual immune cell types, identify new immune cell populations, reveal pathogenic immune cell subsets and transcriptional modules related to pathogenesis, and evaluate immunotherapy efficacy and response.^13^ Furthermore, differential gene expression and intercellular interactions among immune cell types and samples can be evaluated.^14^ In addition to humans, many studies have been conducted on scRNA‐seq of PBMCs in mouse models, providing novel understanding of immune system in healthy and disease conditions.^15^, ^16^, ^17^ These studies not only identified immune cell types, their interactions, and regulatory molecular mechanisms, but also identified potential targets for immune‐related disease therapy.^9^, ^10^ In this study, we compared the cellular taxonomy of PBMCs in 12 species, revealing the conserved and divergent patterns of cellular crosstalk and genetic regulatory networks among multiple species.

## 2 RESULTS

### 2.1 Single‐cell transcriptomic profiles of PBMCs

**FIGURE 1:**
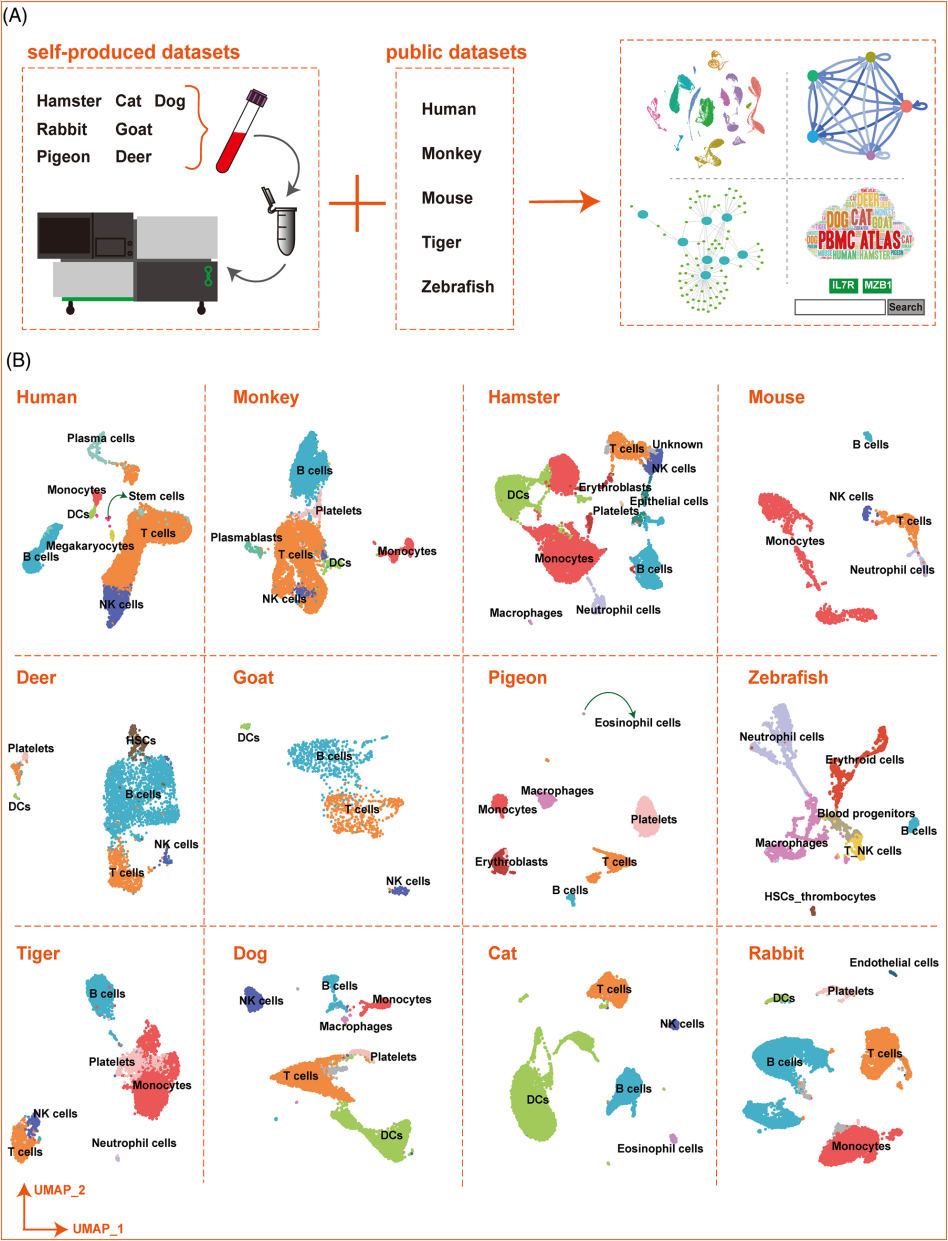
Single‐cell transcriptome atlas of PBMCs for 12 species. (A) Illustration of the overall project design. (B) UMAP plots showing single cell transcriptome atlas of 12 species. Dots with colours represent different cell types which were indicated above

To enable cross‐species comparison of the molecular mechanisms governing immune cell heterogeneity and compositions, we first isolated fresh PBMCs from individuals of seven species: cat (*Felis catus*), dog (*Canis lupus familiaris*), rabbit (*Oryctolagus cuniculus domesticus*), hamster (*Mesocricetus auratus*), deer (*Cervus nippon*), goat (*Capra aegagrus hircus*), and pigeon (*Columba livia domestica*). Using scRNA‐seq and filtering out doublets and low‐quality cells (see Section 5), we obtained high‐quality scRNA‐seq data of 50 478 cells. We next integrated five publicly available PBMC scRNA‐seq datasets: that is, human,^18^ tiger^19^ monkey,^20^ mouse,^21^ and zebrafish^22^ (Supporting information 1a), from which single‐cell transcriptome data of 27 479 cells were obtained for further analyses (Figure 1A).

**FIGURE 2:**
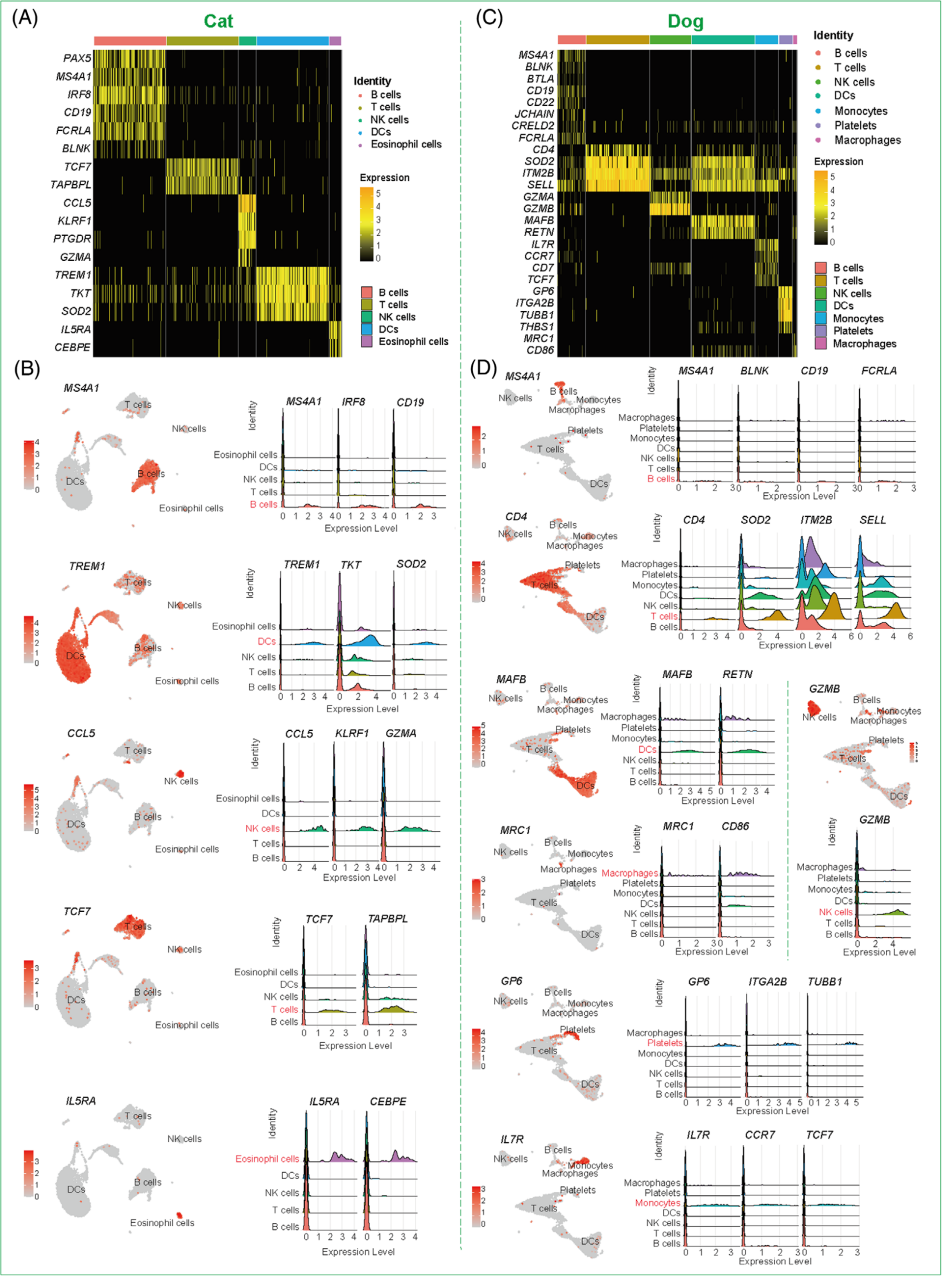
The cellular heterogeneity and compositions of PBMCs in cat and dog. (A,C) Heatmap plots showing a series of genes expressions in various annotated cell types of cat (A) and dog (C). The colours indicated the percentage of cells that showed gene expression levels within each cell type. (B,D) Feature plots and ridge plots showing the gene expression pattern of cat (B) and dog (D) respectively in different cell types

We first humanized the homologous genes for all nonhuman species and performed unsupervised clustering using the top variable genes. We identified five main types of immune cells, that is, T cells, B cells, natural killer (NK) cells, monocytes, and DCs based on the specific expression of cell‐type marker genes (Figure 1B and Figure S1, S2, Supporting information 1b). Cell‐type identity was also confirmed using gene ontology (GO) functional enrichment analysis of differentially expressed genes (DEGs) (Supporting information 2 and 3). For example, in the cat, B cells were annotated based on the high expression of markers *CD19*, *IRF8*, and *MS4A1*; T cells were characterized by the specific expression of *TCF7* and *TAPBPL*; NK cells were identified by enrichment of *CCL5*, *GZMA*, and *KLRF1*; and DCs were characterized by enrichment of *TREM1*, *TKT*, and *SOD2* (Figure 2A,B). In the dog, B cells were annotated based on the high expression of markers *CD19*, *BLNK*, *FCRLA*, and *MS4A1*; T cells were characterized by the specific expression of *CD4*, *SOD2*, *ITM2B*, and *SELL*; NK cells were identified via enrichment of *GZMB*; DCs were confirmed by enrichment of *MAFB* and *RETN*; and monocytes were identified by the expression of *IL‐7R*, *TCF7*, and *CCR7* (Figure 2C,D). The specific expression patterns of these molecules successfully identified the different cell types and provided a molecular basis for exploring the physiological functions of the respective immune systems (Figure S3). In both cat and dog, functional analysis of DEGs in the five main immune cell types indicated that they were primarily related to regulation of innate immune response, regulation of immune effector process, neutrophil activation involved in immune response, immune response‐activating signal transduction, and immune response‐activating cell surface receptor signaling pathway (Figure S3).

### 2.2 Conservation of PBMC connectomes

**FIGURE 3:**
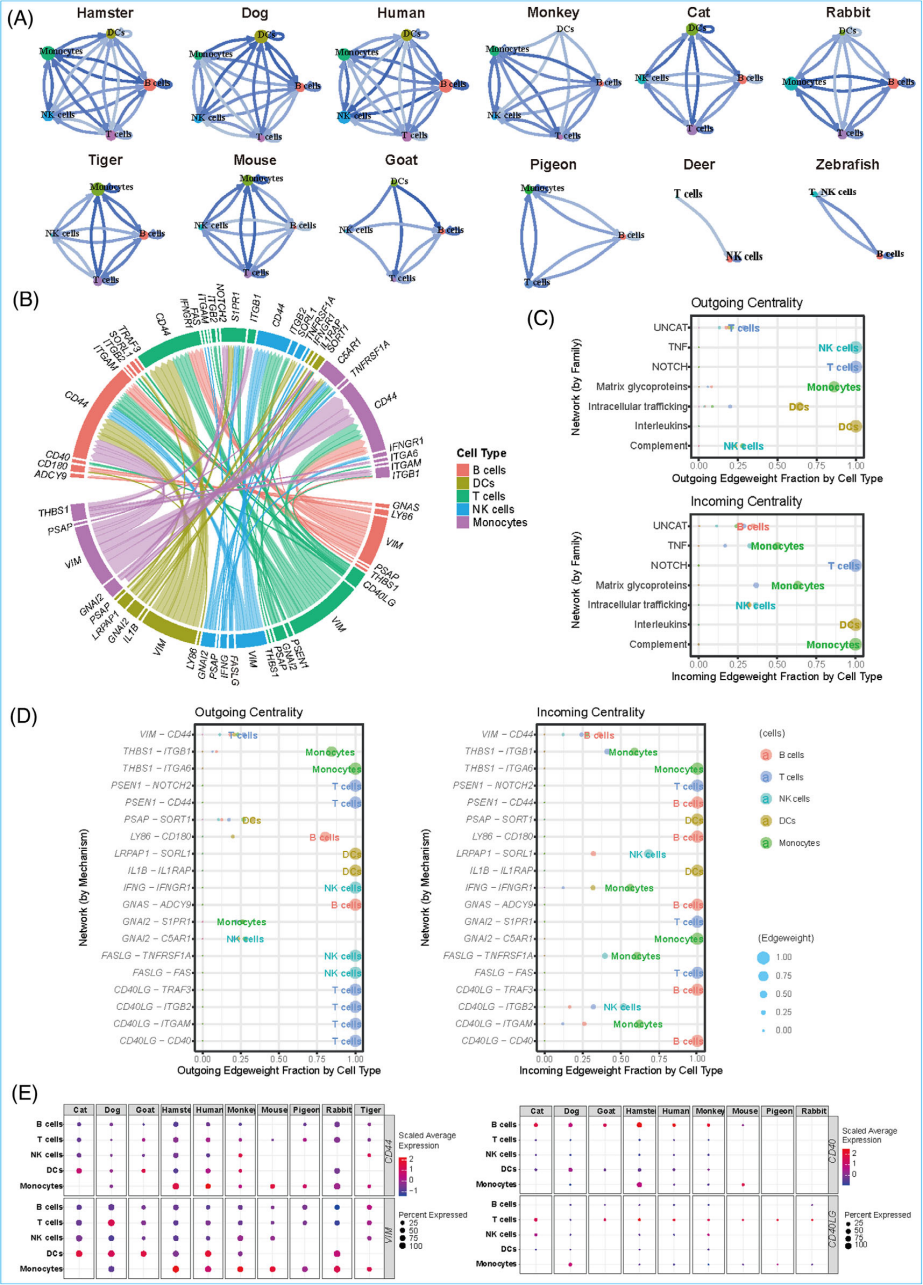
Cross species conserved PBMCs cellular connectomes. (A) Communication network of receptor–ligand pairs between five immune cell types including B cells, T cells, NK cells, DCs, and monocytes. Cell types were represented by coloured node, of which the size was proportional to the sum of receptor–ligand pairs between this node and all other nodes. The edge colour was proportional to the number of receptor–ligand pairs between two connected nodes. (B) Circos plot of cross species conserved connectome. Receptors and ligands were displayed near the upper and lower half circle respectively. (C) Centrality analysis of the conserved connectomes grouped by modes (signalling families). In the centrality plot, the outgoing means sending and incoming means receiving, which refers to quantitative metrics of how ‘connected’ a given edge is to other edge. (D) Centrality analysis of the conserved connectomes grouped by mechanism (ligand–receptor pair). (E) Dot plots showing the co‐expression of two ligand–receptor gene pairs (*VIM*–*CD44*, *CD40LG*–*CD40*) in five immune cells of indicated species

To identify potential cellular interactions, we constructed a ligand receptor mediated communication network of the above five immune cell types for each species using the Connectome R package^23^ (Figure 3A, Supporting information 4). Based on the interaction network data, we further analysed the relationship between ligand–receptor pairs in the different cell types. For example, CD4OLG was identified as a ligand of T cells, and was able to interact with its receptors CD40, TRAF3, and ITGB2 on B cells (Figure 3B). For target analysis of network centrality, these communication pairs were generally classified into seven signalling modalities (i.e., tumour necrosis factor (TNF), NOTCH, matrix glycoproteins, intracellular trafficking, interleukins, complement, and uncategorized) (Figure ).

We further identified connectivity among immune cells. In total, 214 pairs of cell–cell connections were conserved among the five main immune cells in the 12 species (Supporting information 4 m). Among them, there were 19 ligand–receptor pairs showing pan‐conserved immune cell interactions. In addition, ligands related to the *VIM* gene appeared most frequently, and receptors related to the *CD44* gene appeared most frequently, with most belonging to the uncategorized signalling modality (Figure 3D, Supporting information 4 n). In addition to immune cell crosstalks, we explored the conservation of cell–cell crosstalks in different species. Several interactions between immune cells in PBMCs, such as the VIM ligand and CD44 receptor, were commonly expressed in the DCs and T cells of human, monkey, hamster, dog, cat, rabbit, and goat. The CD40LG and CD40, CD40LG and TRAF3, CD40LG and ITGB, were common interactions pairs in T cells–B cells among human, monkey, hamster, mouse, tiger, dog, and cat. The GNAI2–C5AR1 ligand–receptor pair was expressed in the monocytes of six species (human, monkey, hamster, mouse, rabbit, and dog), and categorized as complement signalling modality. Additionally, the PSAP–SORT1 ligand–receptor pair was expressed in the DCs of five species (human, hamster, cat, rabbit, and dog) and belonged to the intracellular trafficking signalling modality (Figure 3E, Figures S4,5).

### 2.3 Conservation of PBMC regulomes

**FIGURE 4:**
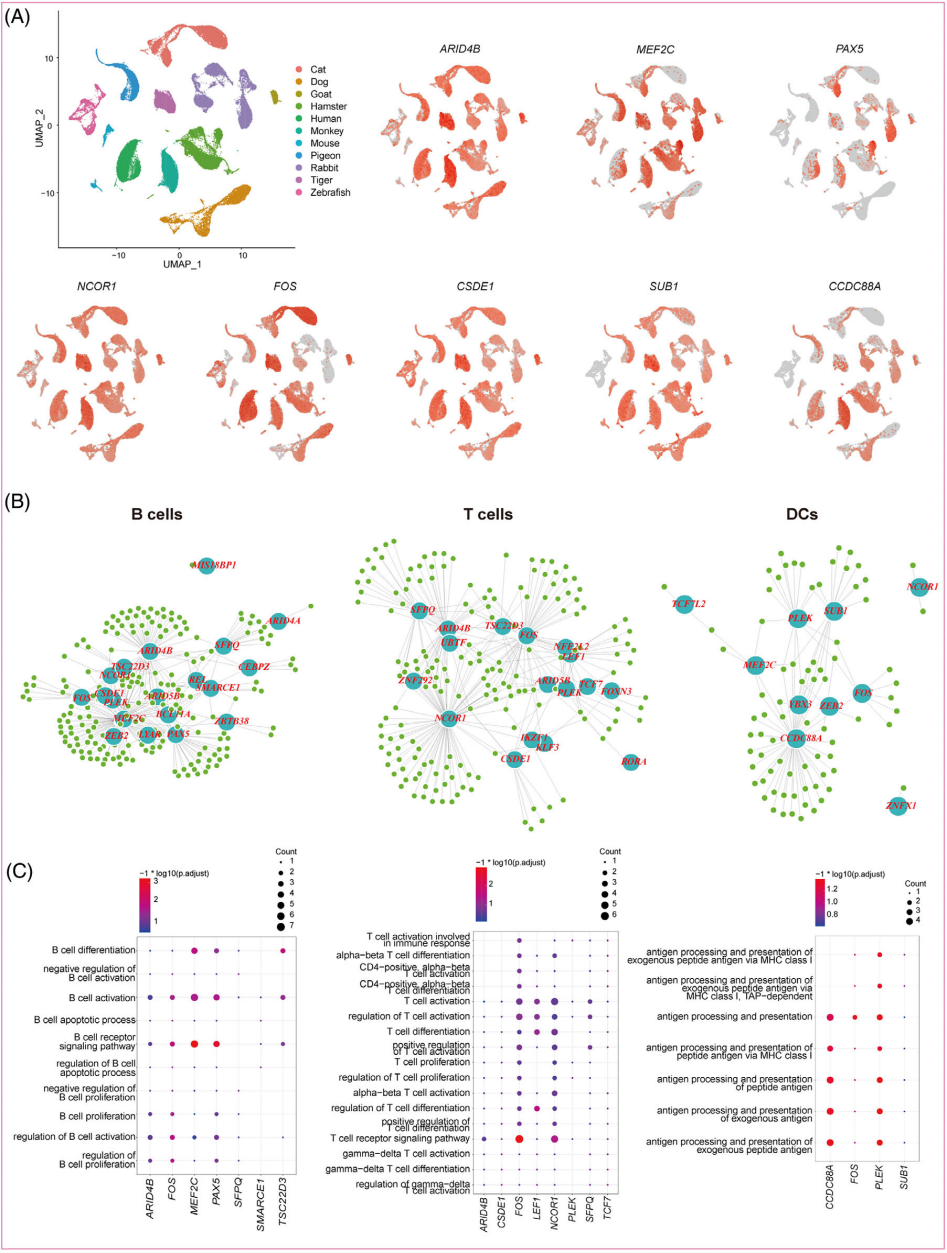
Cross species conservation of regulomes in PBMCs. (A) Feature plots showing high expressions of a series of TFs cross species. A big dataset was merged by 12 species datasets and the different colours indicated different species. (B) Conserved genetic regulatory networks in B cells, T cells and DCs. TF–target interactions with a weight (linkList) value ≥ 0.01 of specific cell type in all species was performed, and the frequency of each pair in each species was counted as SpeciesNumber to evaluate its conservation level. If the SpeciesNumber was ≥ 4, then the TF–target interactions were considered as conserved. Light blue nodes represent regulators, green nodes represent corresponding target genes. Edge width is proportional to weight of regulation, and node size is proportional to the number of target genes of regulator. (C) GO term enrichment related to cellular functions of predicted target genes in B cells, T cells and DCs. Dot colour represents significant level of enrichment analysis and dot size is proportional to the count of target genes classified in GO terms

To explore the regulatory mechanisms underlying the immune system development in the light of evolution, the PBMC genetic regulatory networks were predicted for the 12 species (Figure 4A, Supporting information 5). Subsequently, we analysed the regulatory network of the five immune cell types. A variety of TF–target interactions conserved in at least four species were identified (293 in T cells, 324 in B cells, 108 in DCs, 94 in NK cells, and 194 in monocytes) (Figure 4B, Supporting information 6–10). Enrichment of GO terms for predicted target genes indicated that the regulatory functions of these TFs were closely related to immune response processes (Figures 4C and S6). Many regulatory circuits were highly conserved in each cell type among the multiple species (Figure 4C). Specifically, in B cells, the interactions between *MEF2C* and its targets (*LYN*, *ABRACL*, *ARID5B*, *CANX*, *CCT5*, *HSP90B1*, *PAX5*, *PLEK*, *PNISR*, *RALGPS2*, *SNW1*, *VIM*, and *PAN3*) were highly conserved. The interactions between *PAX5* and its target genes (*LYN* and *VCP*) were conserved, and were mainly related to the differentiation, activation, proliferation, and receptor signalling pathway of B cells. In T cells, the regulatory relationships between *NCOR1* and its target genes (*PTPRC*, *ARHGAP15*, *IQGAP1*, *RPL27*, *SCAF11*, and *YTHDC1*), *TCF7* and its target gene (*LEF1*), and *FOS* and its target genes (*PTPN6*, *TMBIM6*, *RPL27*, *HERPUD1*, and *VIM*) were conserved, and were mainly involved in the differentiation, activation, proliferation, and receptor signalling pathway of T cells. In DCs, *CCDC88A* and its target genes (*PSMA3*, *GOLGA4*, *LGALS1*, *PDIA3*, and *SWAP70*) and *PLEK* and its target genes (*ANXA1* and *SGK1*) were mainly related to antigen processing and presentation. In NK cells, the interactions between *SUB1* and its target gene (*NDUFA8*) were conserved (Figure S6, Supporting information 9). In monocytes, the interactions between *PLEK* and its target genes (*B2M*, *CTLA*, and *LGALS3*) were conserved (Figure S6, Supporting information 10).

### 2.4 Integrated online platform for versatile data exploration

**FIGURE 5:**
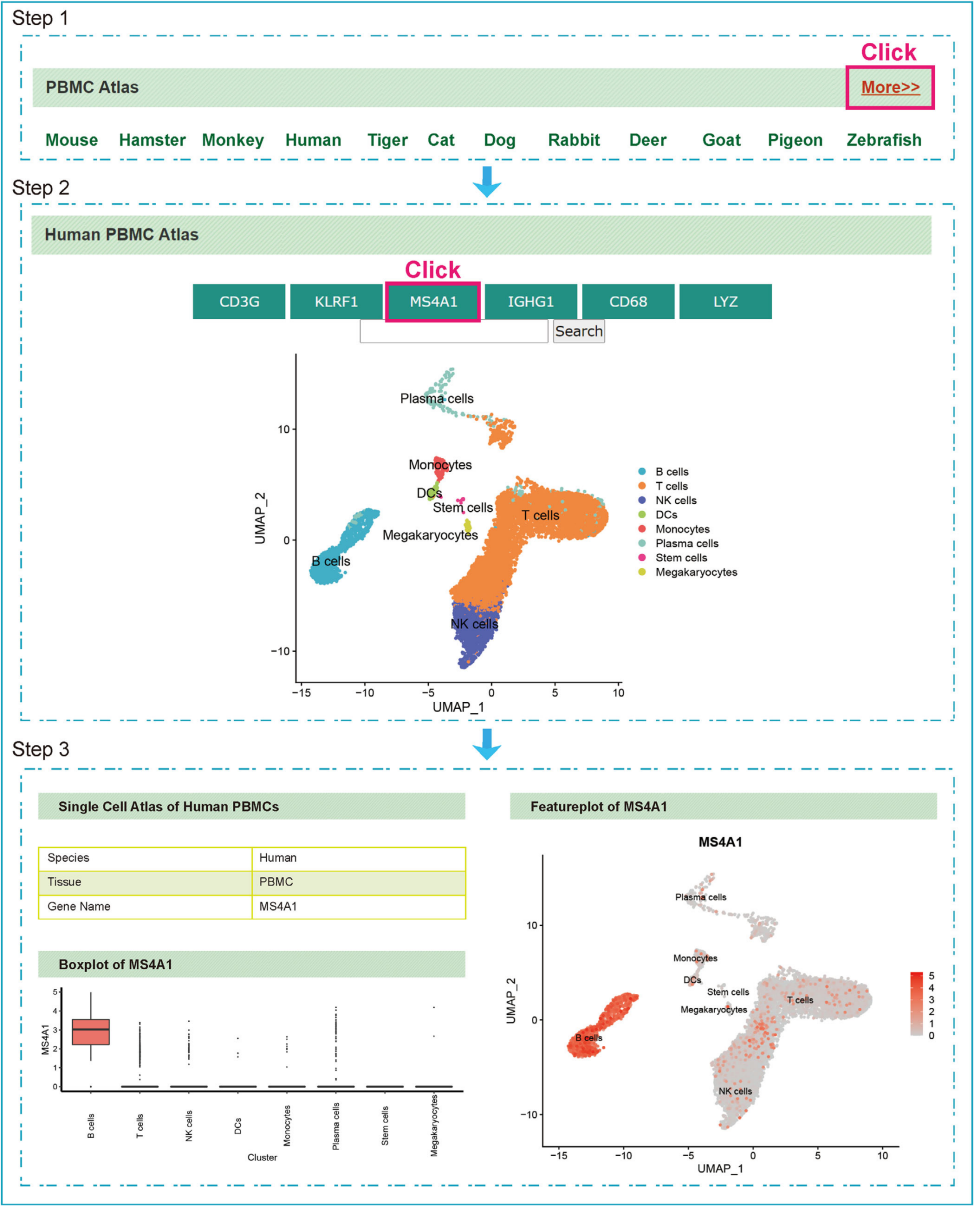
Cross species PBMC atlas website. Based on the scRNA‐seq data of 12 species, a cross‐species PBMC atlas website was generated. It can be used to search for information about gene expression in different species in the form of box plots and feature plots. Additionally, it can be utilized to investigate the conserved genes expression between species at the single‐cell level

To share our data resources, we developed a website named PBMCatlas (http://120.79.46.200:81/Pandora/PBMC.html), allowing users to analyze cell populations of interest and quickly visualize the expression patterns of important genes (Figure 5). This function can assist users to clarify if the input gene is specifically expressed in certain cell types. Thus, PBMCatlas offers a user‐friendly platform to explore our dataset and flexibly access our analysis results, which should facilitate future‐related research.

## 3 DISCUSSION

As the most often used cell model in immunological studies, PBMCs can reflect the dynamic changes in circulating innate and adaptive immune systems and play a vital role in immunotherapy. Here, we used scRNA‐seq analysis to elucidate the heterogeneity of PBMCs, and investigated the conserved cell–cell communications and genetic regulatory networks of major immune cell types across 12 different species.

### 3.1 The cellular heterogeneity and compositions of PBMCs

We surveyed the PBMC atlas by scRNA‐seq for comprehensive analysis. In total, 77 957 cells were derived from the 12 species. After cell clustering analysis, we identified five major immune cell types for each species, including NK cells, B cells, DCs, monocytes, and T cells. The divergences of immune cells across species were also investigated in the previous study.^24^

### 3.2 The conserved cellular crosstalks between immune cells

#### 3.2.1 VIM–CD44 in DCs–T cells

We analysed cell–cell interactions from the classical immune response and focused on DCs–T cells and T cells–B cells, which are involved in antigen presentation processing and adaptive immune response. The interactions between *VIM* and *CD44* in DCs–T cells were identified among 214 pairs of conserved cell interactions in the 12 species. VIM–CD44 is a highly conserved cell–cell crosstalk pair, especially among ligand–receptor pairs in DCs–T cells. *VIM* is expressed in lymphocytes and can interact with other proteins for intercellular signal transduction and can be released as an antigen component of pathogen infection,^25^, ^26^ with bacterial and viral pathogens able to attach to this protein on the host cell surface.^27^ *CD44* is up‐regulated in activated lymphocytes and is involved in various cellular functions, including activation, recirculation, and homing of T‐lymphocytes (T cells are activated, and IL‐2 production is elevated under CD44 stimulation), as well as hematopoiesis, inflammation, and response to bacterial infection.^28^ In our study, the VIM–CD44 interaction is highly conserved among multiple species. VIM on DCs could cooperate with CD44 on T cells, which promotes the antigen presentation and activation of autoreactive T cells. Additionally, the binding of VIM and CD44 activates T cells, triggers activation of a series of possible effector genes, and activates signalling pathways transduction,^29^ while how this signal is transduced and participates in immune responses and host defense requires further study in immune diseases.

#### 3.2.2 CD40LG–CD40 in T cells–B cells

CD40LG–CD40 is among one of the conserved ligand–receptor pairs between T cells and B cells. CD40LG is predominantly expressed in activated CD4^+^ T cells and binds to its ligand (CD40) on the surface of B cells, thereby influencing B cell function.^30^, ^31^ This pair also co‐stimulates T‐cell proliferation and cytokine production,^32^ and enhances the expression of IL‐4 and IL‐10^32^. *CD40LG* deficiency is a severe primary immunodeficiency caused by mutations in the *CD40L* gene, which can lead to T‐cell impairment, B‐cell defects, and susceptibility to opportunistic pathogens.^33^ Mutation of the *CD40* gene can result in type 3 hyper‐IgM immunodeficiency, characterized by an inability to undergo isotype switching, an inability to mount an antibody‐specific immune response, and a lack of germinal centre formation.^34^ Clinical trials have evaluated novel therapeutic approaches targeting the CD40–CD40LG pathway based on T cell–B cell interactions for autoimmune diseases.^35^

### 3.3 The conserved regulatory networks in different immune cells

#### 3.3.1 CCDC88A and its target genes in DCs

Lastly, we investigated the conserved regulatory networks in different immune cells among the 12 species. The coiled‐coil domain containing 88A (*CCDC88A*) gene encoded protein is a kind of coiled‐coil domain containing Girdin family proteins that are activated by Akt and necessary for cytoskeleton remodelling and cell migration.^36^, ^37^, ^38^ CCDC88A accumulates in cell protrusions and contributes to the formation of membrane protrusions and cell migration and invasion,^39^, ^40^ in line with the structural characteristics and functional demands of DCs. In addition, *PSMA3* a target gene of *CCDC88A*, which encodes proteasome, is essential for the generation of a subset of major histocompatibility complex (MHC) class I‐presented antigenic peptides, as well as for the maturation of DCs.^41^ The guanine nucleotide‐binding (G) protein α subunit (Gα)‐interacting vesicle‐associated protein (GIV), protein of *CCDC88A* is most highly expressed in DCs and macrophages and participates in the inhibition of proinflammatory signalling via Toll‐like receptors (TLRs).^42^ These developmental and functional characteristics of DCs may provide new pathways to restore immune tolerance and inhibit self‐antigen presentation processing.

#### 3.3.2 NCOR1, TCF7 and their target genes in T cells

NCOR1 plays an important role in controlling positive and negative selection of thymocytes during T‐cell development.^43^ Furthermore, NCOR1 is considered a novel regulator of immune tolerance and immune cell development,^44^ and shapes the transcriptional landscape, influences the direction of CD4^+^ T cell differentiation, and controls Th1/Th17 effector functions.^45^ In addition, its target gene *PTPRC* (encoded protein tyrosine phosphatase receptor type C), also known as CD45, is essential for T‐cell antigen receptor‐mediated activation, and its downstream regulatory imbalance can result in autoimmunity.^46^, ^47^ *TCF7* is predominantly expressed in T cells and plays a critical role in T‐cell development.^48^, ^49^ Its encoded protein, T cell factor‐1 (TCF‐1), belongs to the T‐cell factor/lymphoid enhancer‐binding factor family. TCF‐1 is highly expressed in naive CD8^+^ T cells but is down‐regulated after differentiation into effector CD8^+^ T cells, and is necessary for the formation of central CD8^+^ T cell memory in response to infection.^50^, ^51^, ^52^ Silencing of Tcf1 facilitates effector CD8^+^ T cell differentiation,^51^ and knockout of *TCF7* in mice results in impaired T‐lymphocyte differentiation.^53^ In addition, its target gene *LEF1*, which encodes lymphoid enhancer binding factor 1, can bind to functionally important sites in the T‐cell receptor‐α enhancer. This is critical for the maturation and development of IL17A‐producing T cells, with its imbalance downstream potentially resulting in autoimmunity.^47^, ^54^

#### 3.3.3 MEF2C, PAX5 and their target genes in B cells

MEF2C binds the active regulatory region to the V(D)J gene in mouse B cell progenitors and human B lymphoblasts, which is essential for lymphatic fate determination.^55^, ^56^ MEF2C has a highly conserved MADS box and MEF2 domain, which contribute to B cell homeostasis.^57^, ^58^ In addition, MEF2C and early B cell factor‐1 together form a co‐regulator, which targets and regulates a subset of B cell‐specific genes.^55^ Various animal models also show that *MEF2C* is important in myeloid leukaemia. Mutations in *MEF2C* are often found in patients with B cell lymphoma, and these mutations are involved in the pathogenesis of abnormal B cell proliferation.^57^, ^59^, ^60^ *LYN* is the target gene of MEF2C, participating in the regulation of B cell differentiation, proliferation, survival, and apoptosis, and plays an important role in maintaining immune self‐tolerance.^61^ It also acts downstream of B cell receptors via the down‐regulation of signalling pathways. As another crucial regulatory gene of B cells, paired box protein 5 (*PAX5*) is necessary for the differentiation of lymphoid progenitor cells into B lymphocyte lineage.^62^ PAX5 regulates transcriptional reprogramming processes by restricting uncommitted progenitors to the B cell pathway, promoting V(H) –DJ(H) recombination, inducing B‐cell receptor signalling, and facilitating development to the mature B‐cell stage.^62^, ^63^ However, PAX5 inhibition is not necessary for stable plasma cell development and antibody secretion, even though it is essential for immunoglobulin G (IgG) production and long‐lived plasma cell increase.^64^ Thus, the role of PAX5 in plasma cell differentiation needs to be further investigated. *LYN* is also a target gene of PAX5, and studies show that both are related to B cell development.^65^, ^66^ Collectively, we identified highly conserved regulomes in the PBMCs of different animal species. Identifying conserved key genes and exploring their functions in multiple species will help improve our understanding of the development, maturation, proliferation, activation, differentiation of immune cells.

In this study, we produced the comprehensive PBMC atlas of 12 species, which holds significance for immunological research. We systematically studied the gene expression profiles and molecular characteristics of each cell type and compared them across species at single‐cell resolution. We also identified key genes and highly conserved cell–cell interactions that play important roles in regulating development and immune response. The PBMC atlas website was constructed, which provides an accessible approach to explore different species datasets. These results provide a systematic resource for understanding immune cell diversity as well as insights into the molecular mechanisms governing conservation of PBMCs across species.

## 4 LIMIATIONS OF THE STUDY

A few limitations of this study were also addressed, which should be investigated by further study. First, the conservation of homologous genes in non‐human species was calculated to identify distinct cell types of PBMCs in different species. However, the non‐traditional species (e.g., deer, rabbit ) showed low ratios of homologous genes to humans, resulting in lower diversity in cell types of PBMCs for these species. Second, when comparing cell‐type specific gene expression across species, the effects of age, sex, and physiological conditions were not considered. Nevertheless, major immune cell types were identified in all these species, and cellular heterogeneity and compositions of PBMCs were characterized. The connectome analysis may be influenced by potential species differences in ligand–receptor interactions or homolog conversion. Last, the aim of this study was to focus on the single‐cell atlas of PBMCs from multiple species, even though mRNA and protein expression in immune cells can show discrepancies when using different methods.^67^, ^68^, ^69^ In addition, the PBMCs datasets for the 12 species were obtained from different platforms, and differences caused by sample processing, scRNA‐seq technical bias and batch effects could impact cell capture and cell‐type classifications, resulting in technical differences. Importantly, despite these limitations, this study provides a key resource of PBMCs for understanding the immune cell‐type diversity as well as insights into the developmental and envolutionary biology of the circulating immune system across species.

## 5 MATERIAL AND METHODS

### 5.1 Ethics statement

The collection and experimental processing of all samples used in this article were strictly carried out according to the ‘‘Guidelines on the Ethical Treatment of Experimental Animals” established by the Ministry of Science and Technology, China. The Institutional Review Board on Ethics Committee of BGI reviewed and approved this study (NOS. BGI‐IRB A20008, BGI‐IRB A20008 T1).

### 5.2 Blood samples collection

Blood samples were obtained from seven animals, including: cat (*Felis catus*), dog (*Canis lupus familiaris*), hamster (*Mesocricetus auratus*), goat (*Capra aegagrus hircus*), rabbit (*Oryctolagus cuniculus domesticus*), pigeon (*Columba livia domestica*), and deer (*Cervus nippon*). The blood samples of deer with related genome assembly were provided by the Institute of Special Animal and Plant Sciences (ISAPS) of the Chinese Academy of Agricultural Sciences. Other bloods samples were obtained from farmers’ markets with permission from the BGI Ethics Committee. The gene expression matrices for five other species (human,^18^ tiger,^19^ monkey,^20^ mouse,^21^ zebrafish^22^) were obtained from published datasets.

### 5.3 Peripheral blood mononuclear cells processing

All operations were performed under sterile conditions. Peripheral blood samples (3 mL) were collected into an EDTA anticoagulant tube, gently reversed 4‐6 times, and fully mixed, and placed at room temperature. Whole blood was diluted with 3 mL of phosphate‐buffered saline (PBS) and transfered in to a 15 mL centrifuge tube. After this, 6 mL of Histopaque‐1077 (Cat. No. 10771‐6X100ml) was slowly added into the 15 mL centrifuge tube, followed by density gradient centrifugation methods to collect peripheral blood mononuclear cells (PBMCs).

### 5.4 Single‐cell RNA‐seq library construction and sequencing

The goat, deer and pigeon PBMCs underwent library construction using DNBelab C Series Single‐Cell Library Prep Set (MGI) and sequenced with BGISEQ‐500 in China National GeneBank (CNGB). Cat, dog, rabbit and hamster PBMCs underwent library construction using a 10X Chromium Next GEM Single cell 3’ Reagent Kits v3.1 following the guidelines provided by the manufacturer and sequenced using NOVAseq 6000 sequencing platform of Illumina.

### 5.5 Cross‐species homologous gene conversion

We refered to the previous methodological section for cross‐species homologous gene transfer and single‐cell RNA‐seq data processing.^19^

### 5.6 Single‐cell RNA‐seq data preprocessing

Genome information used for read alignment was downloaded from the NCBI Assembly (Supporting information 1b). The raw data was processed using Cell Ranger v3.0.2 (10X Genomics) and Seurat.^71^, ^72^

### 5.7 Cell‐type annotation

For annotation of the self‐produced datasets, our annotation used several classic cell‐type markers from the CellMarker database.^73^ The four published datasets used cell‐type markers from corresponding published article. The annotation results are presented in Figure 1B using UMAP plots.

### 5.8 Differentially expressed genes (DEGs) and Gene Ontology (GO) term enrichment analysis

All DEGs for each cell type were identified using the FindAllMarkers function in Seurat (Supporting information 2). The hypergeometric test implemented in the clusterProfiler^74^ package with the compareCluster function (ENTREZID∼celltype, fun = “enrichGO”, “org.Hs.eg.db”, *p*‐value cutoff = 0.05) was used to carry out GO term enrichment analysis (Supporting information 3).

### 5.9 Cellular communication analysis

We applied the Connectome (https://github.com/msraredon/Connectome) R package^23^ for cellular communication analysis. All ligands and receptors data was downloaded from the FANTOM5 database.^75^, ^76^ First, the five major immune cell types (B cells, T cells, NK cells, DCs, monocytes) were extracted from the annotated datasets of the 12 species for further analysis. The connectome networks were then constructed according to the expression of ligands and receptors.

### 5.10 Transcription Factor (TF)‐target interaction analysis

We applied the GENIE3^77^ R package for TF‐target interaction analysis using data from 11 species (cat, dog, goat, hamster, human, monkey, mouse, pigeon, rabbit, tiger, and zebrafish). The human's TF list was downloaded from animalTFDB3.0.^78^ The igraph R package^79^ was used to visualize representative regulatory TFs networks.

## CONFLICT OF INTEREST

The authors declare that there is no conflict of interest.

## Supporting information



Supporting Information

Supporting Information

Supporting Information

Supporting Information

Supporting Information

Supporting Information

Supporting Information

Supporting Information

Supporting Information

Supporting Information

Supporting Information

Supporting Information

Supporting Information

Supporting Information

Supporting Information

Supporting Information
