# Supplementary figures and images for: Molecular mechanisms governing circulating immune cell heterogeneity across different species revealed by single‐cell sequencing
Source: Clin Transl Med. 2022 Jan 29;12(1):e689. doi: 10.1002/ctm2.689 (PMC8800483; doi:10.1002/ctm2.689)

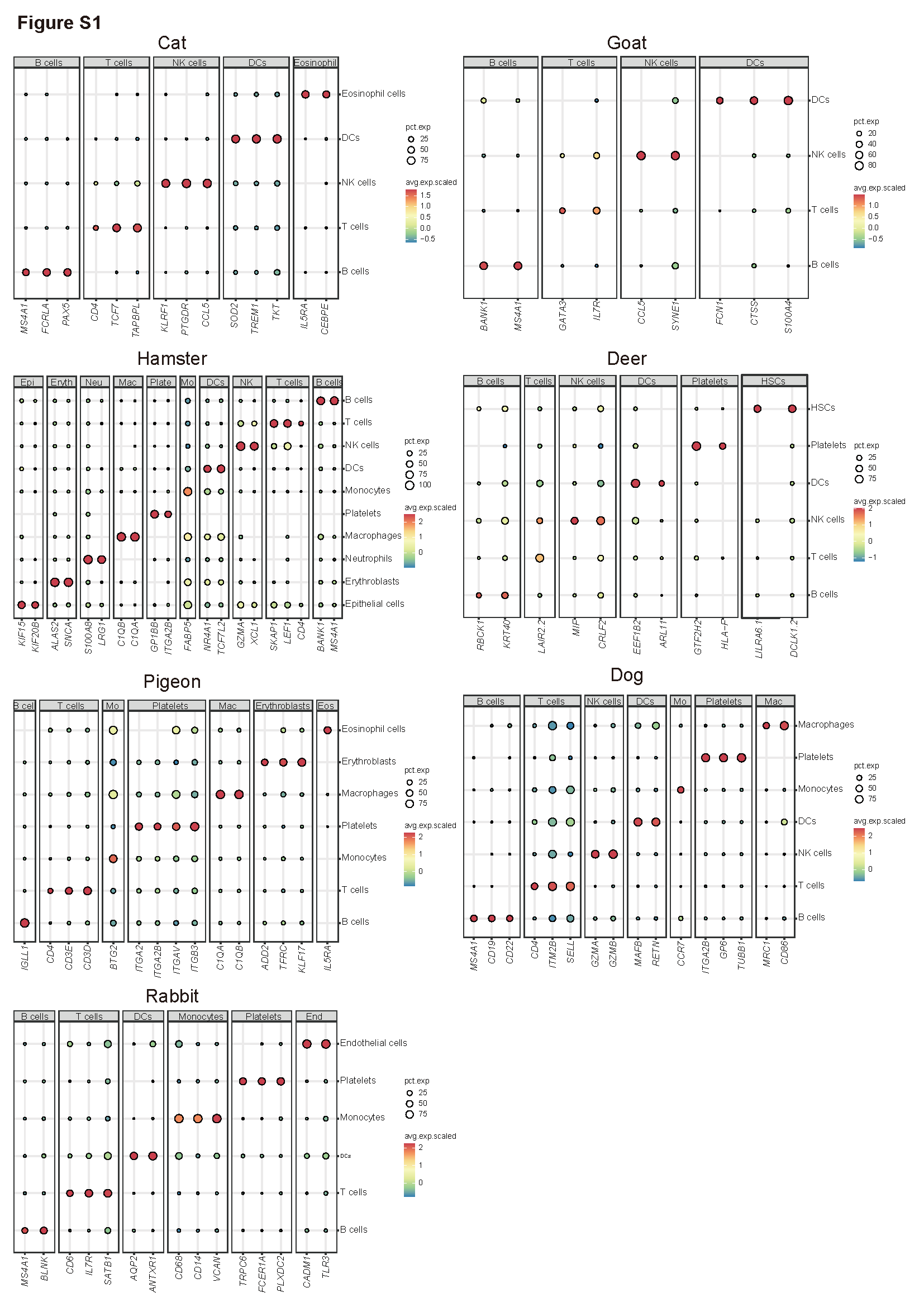

Supplement: Supplementary file 1 — Supporting Information [file CTM2-12-e689-s013.png]

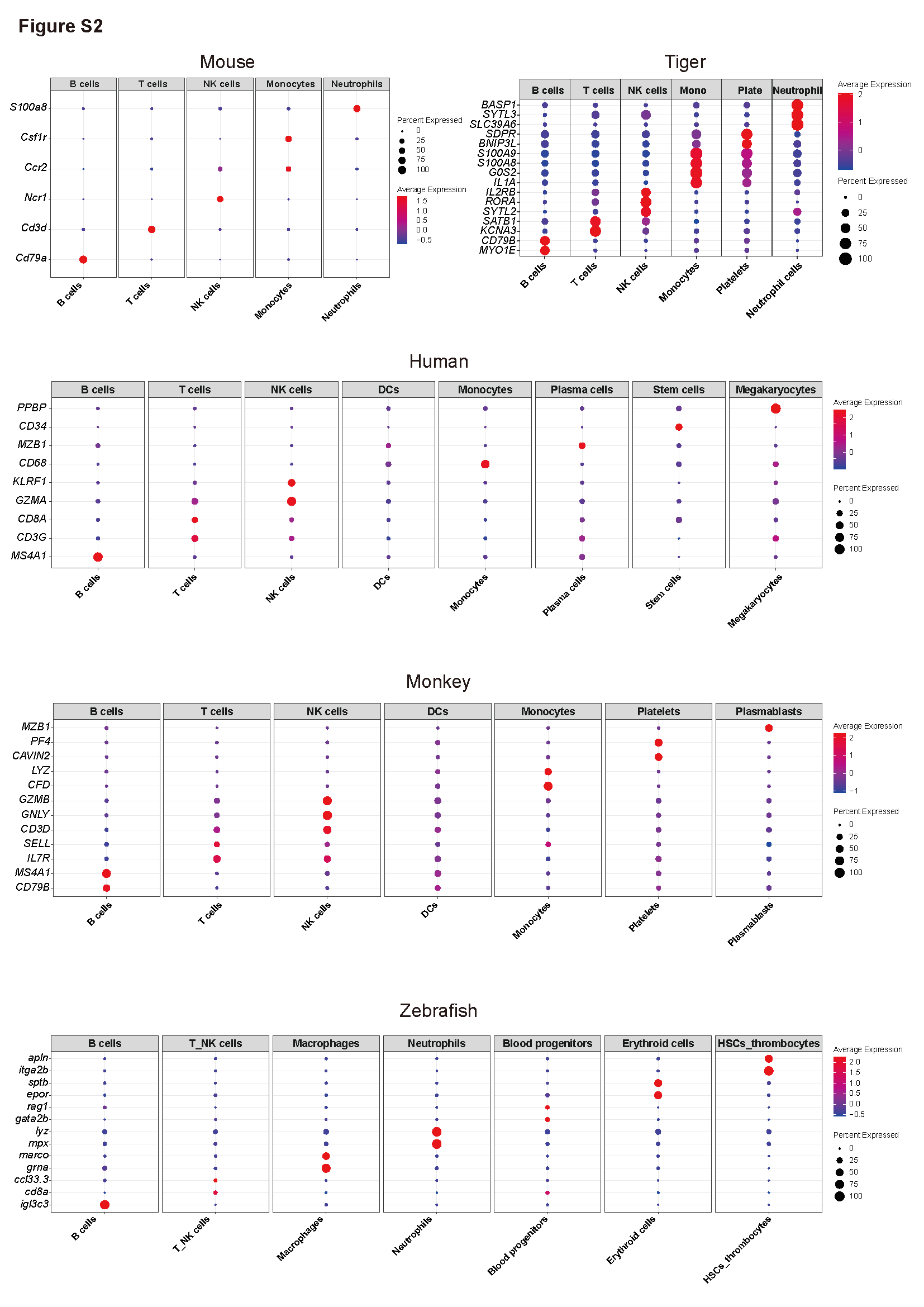

Supplement: Supplementary file 2 — Supporting Information [file CTM2-12-e689-s009.png]

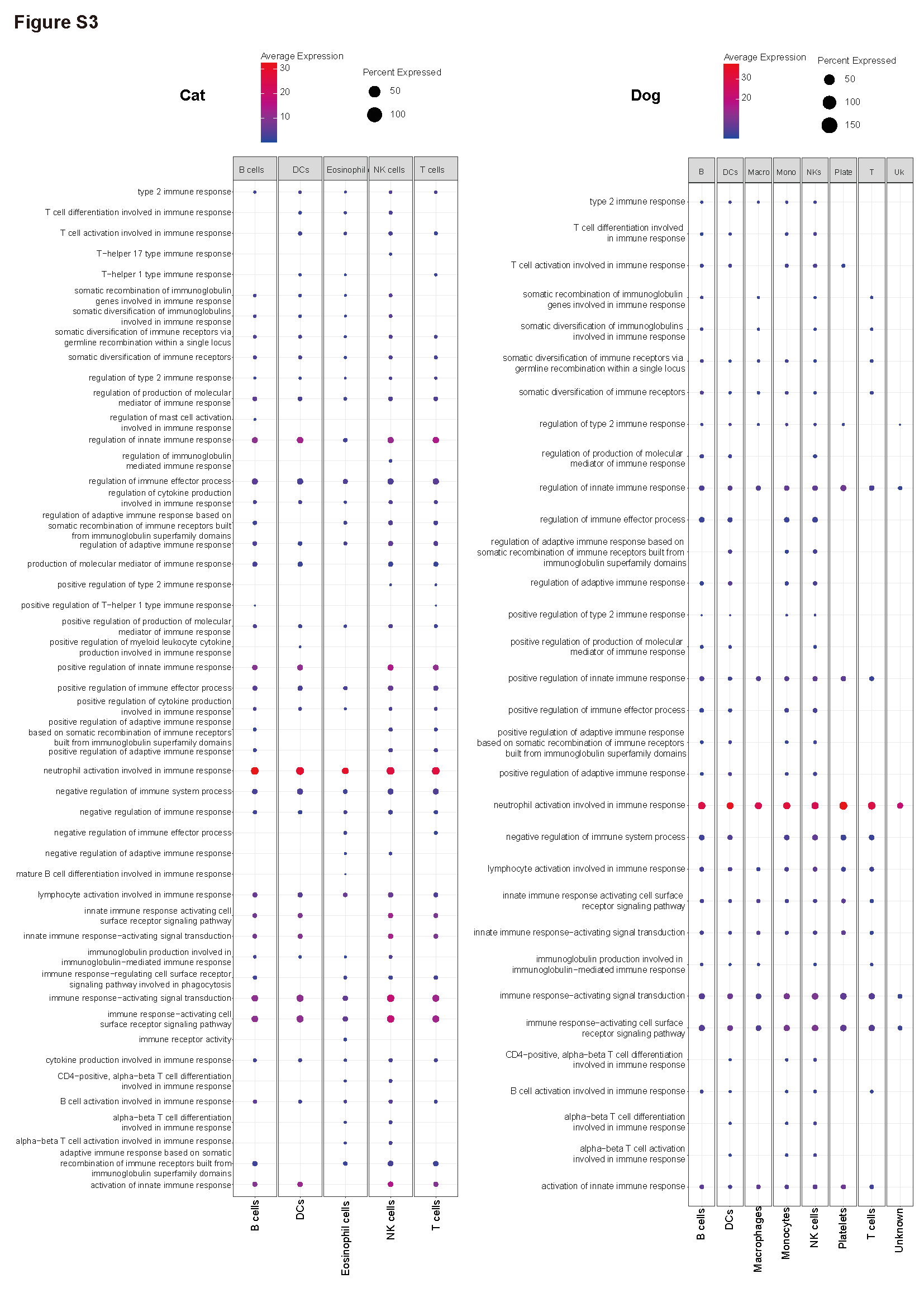

Supplement: Supplementary file 3 — Supporting Information [file CTM2-12-e689-s015.png]

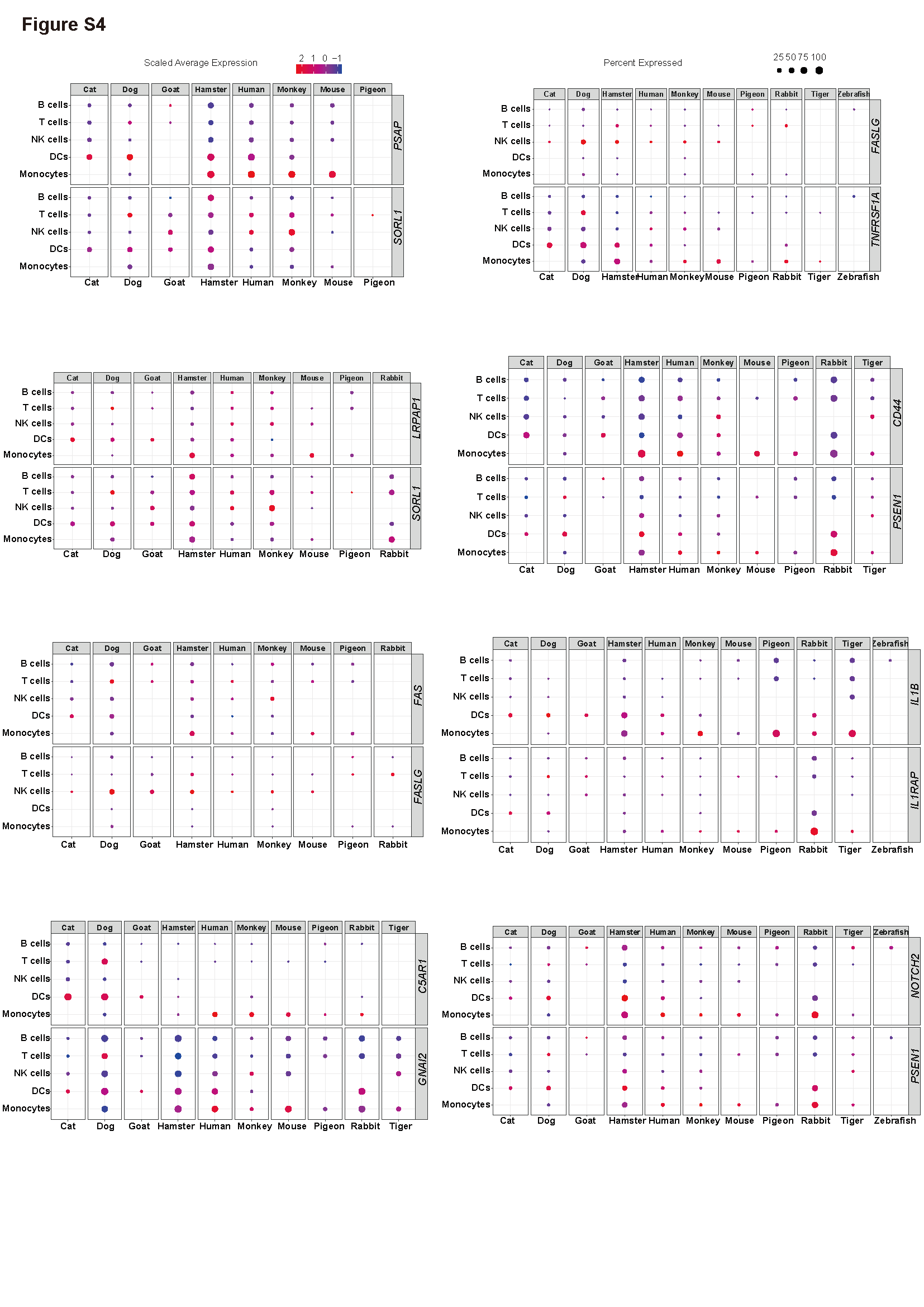

Supplement: Supplementary file 4 — Supporting Information [file CTM2-12-e689-s002.png]

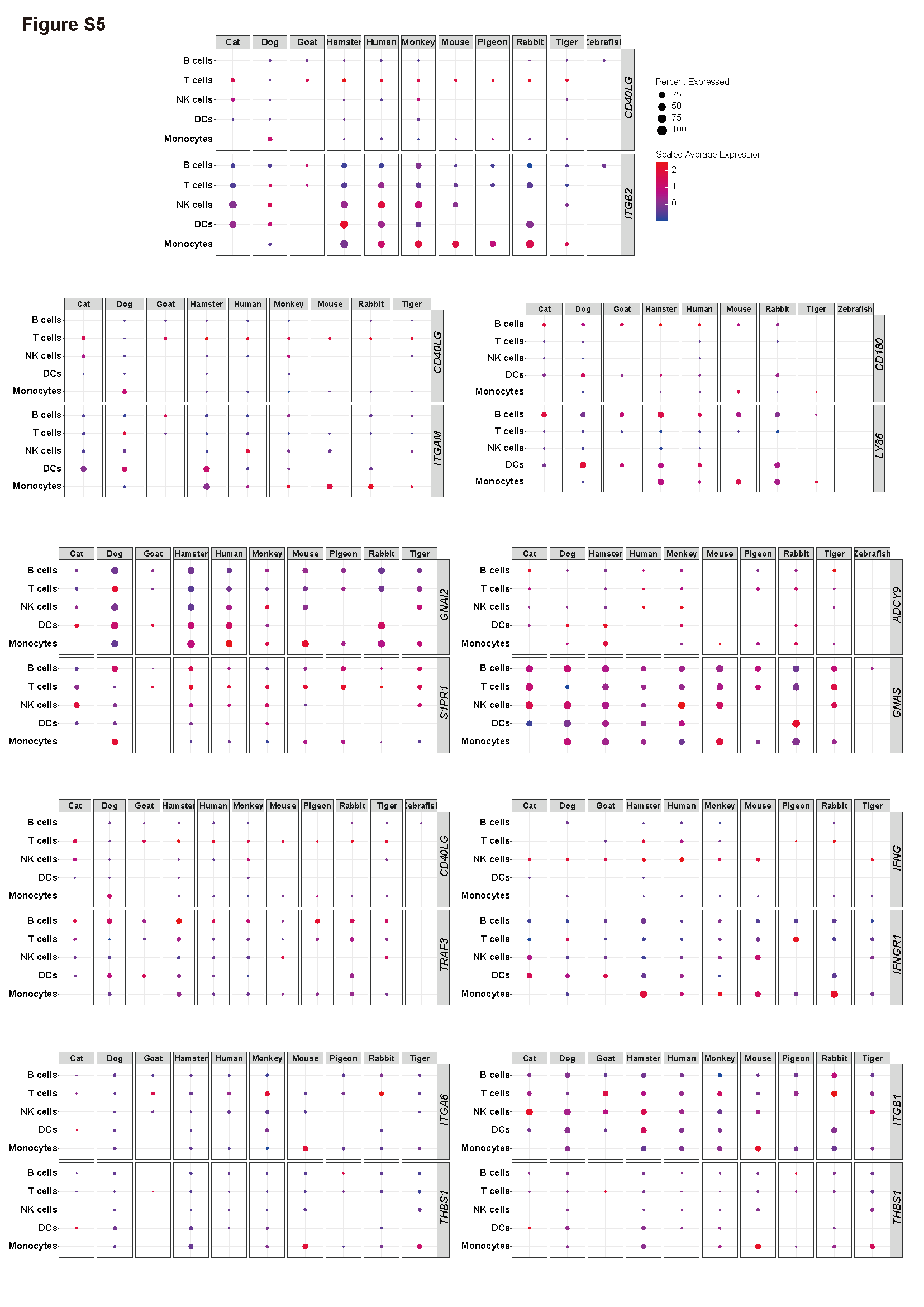

Supplement: Supplementary file 5 — Supporting Information [file CTM2-12-e689-s006.png]

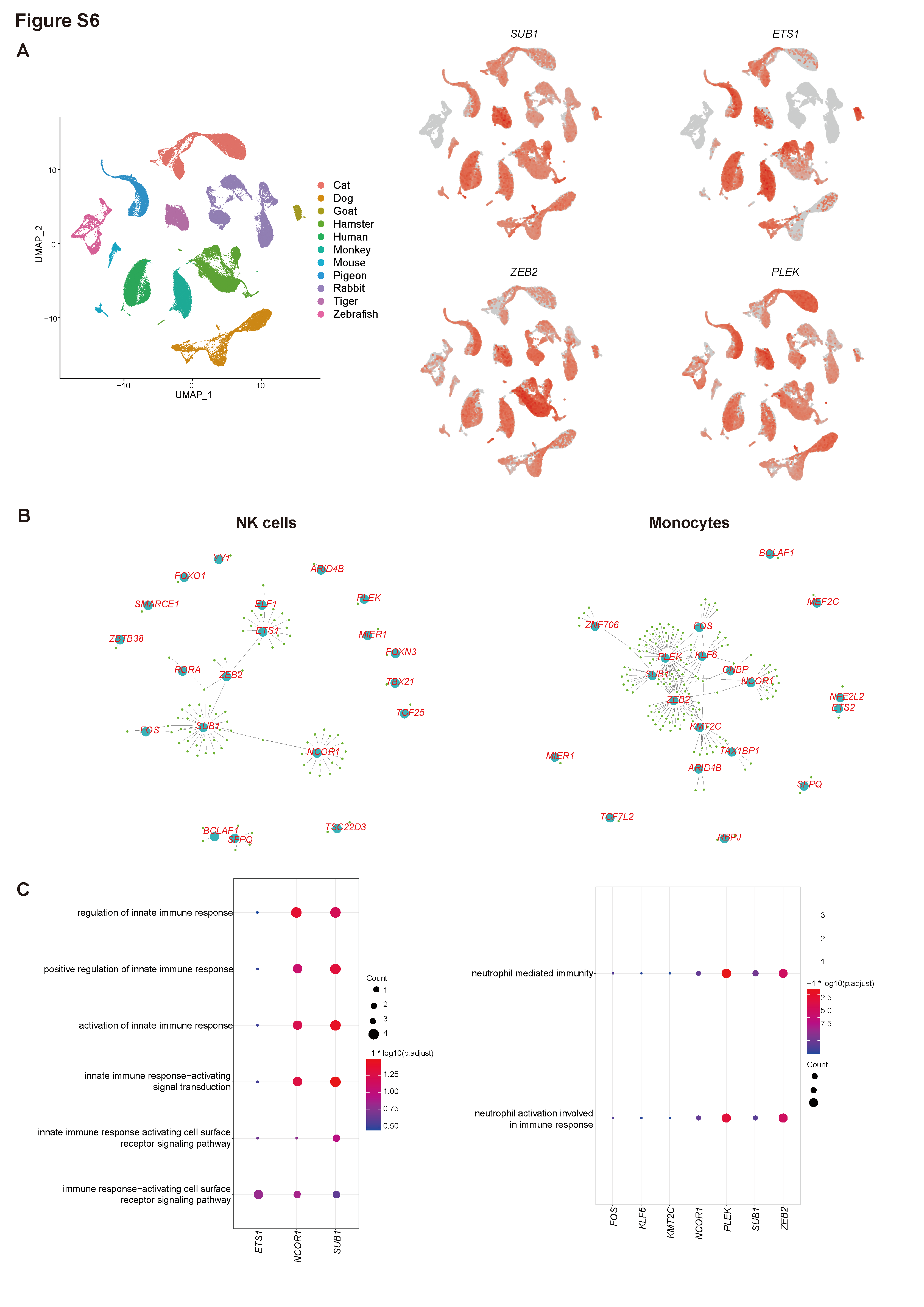

Supplement: Supplementary file 6 — Supporting Information [file CTM2-12-e689-s008.png]
